# Mutually exclusive genetic interactions and gene essentiality shape the genomic landscape of primary melanoma

**DOI:** 10.1002/path.6019

**Published:** 2022-11-09

**Authors:** Sofia Birkeälv, Mark Harland, Larissa Satiko Alcantara Sekimoto Matsuyama, Mamun Rashid, Ishan Mehta, Jonathan P Laye, Kerstin Haase, Tracey Mell, Vivek Iyer, Carla Daniela Robles‐Espinoza, Ultan McDermott, Peter van Loo, Marieke L Kuijjer, Patricia A Possik, Silvya Stuchi Maria Engler, D Timothy Bishop, Julia Newton‐Bishop, David J Adams

**Affiliations:** ^1^ Wellcome Sanger Institute Wellcome Trust Genome Campus Cambridge UK; ^2^ Division of Haematology and Immunology University of Leeds School of Medicine Leeds UK; ^3^ Department of Clinical and Toxicological Analyses, School of Pharmaceutical Sciences University of Sao Paulo Sao Paulo Brazil; ^4^ The Francis Crick Institute London UK; ^5^ Laboratorio Internacional de Investigación sobre el Genoma Humano Universidad Nacional Autónoma de México, Campus Juriquilla Santiago de Querétaro Mexico; ^6^ Centre for Molecular Medicine Norway (NCMM), Nordic EMBL Partnership, Faculty of Medicine University of Oslo Oslo Norway; ^7^ Department of Pathology and Leiden Center for Computational Oncology Leiden University Medical Center Leiden the Netherlands; ^8^ Division of Experimental and Translational Research Brazilian National Cancer Institute Rio de Janeiro Brazil

**Keywords:** melanoma, gene essentiality, genetic epistasis, genomic landscape, CRISPR screening, interferon regulatory factor 4 (*IRF4)*, genome sequencing, somatic mutation, driver genes, primary cancer

## Abstract

Melanoma is a heterogenous malignancy with an unpredictable clinical course. Most patients who present in the clinic are diagnosed with primary melanoma, yet large‐scale sequencing efforts have focused primarily on metastatic disease. In this study we sequence‐profiled 524 American Joint Committee on Cancer Stage I–III primary tumours. Our analysis of these data reveals recurrent driver mutations, mutually exclusive genetic interactions, where two genes were never or rarely co‐mutated, and an absence of co‐occurring genetic events. Further, we intersected copy number calls from our primary melanoma data with whole‐genome CRISPR screening data to identify the transcription factor interferon regulatory factor 4 (*IRF4*) as a melanoma‐associated dependency. © 2022 The Authors. *The Journal of Pathology* published by John Wiley & Sons Ltd on behalf of The Pathological Society of Great Britain and Ireland.

## Introduction

Melanoma remains one of the most curable malignancies when diagnosed at early stages yet is difficult to treat in patients who develop metastases. In European‐descent populations, the most common type is non‐acral cutaneous melanoma, which can be further subdivided into superficial spreading, nodular and lentigo maligna melanoma [1, 2]. These melanoma subtypes are causally related to ultraviolet (UV) light exposure since a history of sunburn and accumulative UV damage has been linked to disease development [3]. Individuals at increased risk of melanoma include those carrying germline alleles of common genetic variants of pigment‐related genes such as *MC1R* [4, 5, 6], whereas high penetrance variants affecting genes such as those that regulate telomere length and stability (*TERT, POT1*) and cell cycle control/senescence (*CDKN2A, CDK4*) have also been implicated [7, 8, 9, 10, 11]. Another subtype of cutaneous melanoma that is not associated with UV light exposure is acral melanoma, which forms on non‐hair‐bearing (glabrous) skin such as the palms of the hands and soles of the feet and is proportionally more common in people of Latin American, Asian and African descent [12, 13]. Melanomas forming on mucosal surfaces, such as the wet mucosa of the oral cavity or anogenital tract, are infrequently observed but are a further subtype notable for their late diagnosis and poor prognosis [14]. Notably, large‐scale efforts to sequence cutaneous melanomas from sun‐exposed sites have defined a somatic landscape that is replete with UV‐associated C‐to‐T mutations, whereas rarer melanoma subtypes, such as acral and mucosal melanoma, appear to carry fewer single nucleotide variants but have genomes that show extensive copy number gains and losses [15, 16, 17]. Thus, both germline and somatic variants can influence melanoma incidence and presentation, as well as the somatic genetic landscape of the disease.

The aforementioned sequencing efforts have revealed many of the key driver mutations that promote tumour development. For example, it is well established that activation of the mitogen‐activated protein kinase (MAPK) pathway is critical for melanoma development, with the mutation of genes such as *BRAF*, *NRAS* and *NF1* defining around three‐quarters of non‐acral cutaneous melanoma cases [17]. Somatic alterations affecting genes such as *CDKN2A*, regulators of cell growth such as genes in the PI3‐kinase pathway (*PTEN*) and telomere maintenance genes (*TERT*), are also frequently observed. Intriguingly, although some of these core pathways are also altered in acral and mucosal melanoma, this may be via different constellations of driver genes and genetic events [16, 18], again distinguishing the different subtypes of melanoma as different diseases.

The eighth edition of the American Joint Committee on Cancer (AJCC) guidelines for melanoma classifies the disease into prognostic stages ranging from I to IV, with associated substages ranging from A to D, based on tumour thickness, level of ulceration and presence/absence of metastasis [19, 20]. Most patients presenting with Stage I or II disease are cured surgically with no further treatment required. A subset of these patients, however, will relapse with metastases. Importantly, we are currently unable to identify those patients at greatest risk of relapse and, thus, those individuals who may benefit from adjuvant or neo‐adjuvant therapies earlier in the disease course.

Notably, as mentioned earlier, large‐scale sequencing efforts of melanoma have revolutionized our understanding of the disease, yet few primary melanomas have been analysed. Primary melanomas, particularly those from thin Stage I cases, may be less than 1 mm thick. Therefore, the analysis of these lesions has historically been technically challenging. In this study, we analyse 524 primary melanoma cases and present a comprehensive evaluation of the somatic alterations found in this disease so as to define the genetic landscape of melanoma at its earliest stages. We reveal a heterogenous tumour architecture alongside the discovery of novel candidate driver genes, new hotspot mutations and promoter variants, as well as mutually exclusive genetic interactions, that help refine our understanding of disease development.

## Materials and methods

### Human ethics statement

The Leeds Melanoma Cohort (LMC) Study is a prospective cohort recruited from a geographically defined area of the UK in the period 2000–2012, Research Ethics Committee (REC) Reference No. 01/3/057 [21] and NIHR/CPMS ID – 15064 (Central Portfolio Management System). Recruitment was on average 5 months after diagnosis [22]. Informed consent was obtained from all subjects, and the experiments conformed to the principles set out in the World Medical Association Declaration of Helsinki and the Department of Health and Human Services Belmont Report. Genetic analysis was further approved by the Human Ethics Committee of the Wellcome Sanger Institute.

### Samples and DNA extraction

Formalin‐fixed, paraffin‐embedded (FFPE) tumour blocks were sampled horizontally using a 0.6‐mm microarray needle from the invasive tumour. The sample site was selected by a single observer to consistently collect material from the least inflamed, least necrotic area of the tumour, as previously described by Nsengimana *et al*. [23]. Cores were processed to extract DNA using the Qiagen AllPrep DNA/RNA FFPE kit (Qiagen, Hilden, Germany). Germline DNA was extracted from peripheral blood using the Nucleon2 kit (Cytiva, Marlborough, MA, USA). Table 1 provides a summary of the clinical characteristics of the patients/samples that were sequenced as part of this study. Missing values were excluded from this output.

**Table 1:** Clinical characteristics of participants in Leeds melanoma cohort [21], whose primary tumours were sequenced as part of this project.

	Overall (*n* = 524)	Alive (*n* = 351)	Death	
			Melanoma (*n* = 141)	Non‐melanoma (*n* = 32)
Sex				
Female	263 (50%)	193 (55%)	59 (42%)	11 (34%)
Male	261 (50%)	158 (45%)	82 (58%)	21 (66%)
Age (years)				
Mean (SD)	57 (12)	55 (12)	60 (12)	66 (8.1)
AJCC stage*				
I	167 (32%)	133 (38%)	25 (18%)	9 (28%)
II	253 (48%)	168 (48%)	67 (48%)	18 (56%)
III	97 (19%)	47 (13%)	46 (33%)	4 (12%)
Breslow thickness* (mm)				
Mean (SD)	3.0 (2.4)	2.6 (1.9)	4.1 (3.2)	3.3 (2.5)
Ulceration*				
No	289 (55%)	215 (61%)	61 (43%)	13 (41%)
Yes	169 (32%)	89 (25%)	67 (48%)	13 (41%)
Mitotic rate* (mitoses/ per mm^2^)				
<1	66 (13%)	52 (15%)	11 (8%)	3 (9%)
≥1	402 (77%)	264 (75%)	116 (82%)	22 (69%)
Tumour‐infiltrating lymphocytes*				
Absent	83 (16%)	57 (16%)	24 (17%)	2 (6%)
Yes (Unclassified)	47 (9%)	36 (10%)	6 (4%)	5 (16%)
Non‐brisk	215 (41%)	132 (38%)	72 (51%)	11 (34%)
Brisk	77 (15%)	61 (17%)	13 (9%)	3 (9%)
Mutational subtype^†^				
*BRAF*	205 (39%)	138 (39%)	57 (40%)	10 (31%)
*NRAS*	148 (28%)	97 (28%)	38 (27%)	13 (41%)
*NF1*	32 (6%)	22 (6%)	8 (6%)	2 (6%)
WT	139 (27%)	94 (27%)	38 (27%)	7 (22%)
Mutation load^‡^				
Mean (SD)	5.1 (7.2)	5.2 (6.7)	4.5 (7.6)	6.9 (10)
Relapse				
No	333 (64%)	304 (87%)	0 (0%)	29 (91%)
Yes	191 (36%)	47 (13%)	141 (100%)	3 (9%)
Immunotherapy				
No	507 (97%)	346 (99%)	129 (91%)	32 (100%)
Yes	17 (3%)	5 (1%)	12 (9%)	0 (0%)

* Missing values were excluded from table output except in column %.

^†^ NRAS codons 12, 13 and 61 and *BRAF* exon 15 somatic mutations are shown along with mutations in *NF1*. These data were derived from the panel sequencing performed as part of this study.

^‡^ Mutation load is defined as the number of nonsynonymous mutations per megabase (MB) of genomic space sequenced.

### Classification of tumours

We classified tumours as non‐acral cutaneous, acral, mucosal or rare sites, which included ano‐uro‐genital cases, such as those arising on vulval, vaginal, anal and penile locations but not defined as being of mucosal origin (supplementary material, Table S1). Superficial spreading, nodular and lentigo maligna subtypes of melanoma were grouped together.

### Sequencing and mutation calling

Targeted capture was performed with Agilent (Santa Clara, CA, USA) SureSelectXT probes using a custom design (supplementary material, Table S2). These baits captured 6.173 MB of genomic sequence (ELID: S3065404). The genes covered by baits in this design included established melanoma and solid tumour driver genes, including clinically actionable genes [17, 24], and the promoter region of several genes previously found to be mutated in melanoma, including *DPH3*, *TERT*, *NDUF89*, *SDHD* and *NFKBIE* [25]. Designs covered all exons of driver genes. Full details are provided in the first authors' published thesis [26]. Sequencing was performed using the Illumina (San Diego, CA, USA) HiSeq4000 platform using 75 bp paired reads. Reads were mapped to the human reference genome assembly GRCh37d5 using BWA‐mem version 2.0.72 [27]. Somatic mutation calling was performed using CaVEman version 1.11.2 [28] and Pindel version 2.2.2 [29]. For 59 tumours there was no matched normal, so one normal sample (PD36169b) was selected as the matched normal sample for the somatic variant calling of these samples. This sample was selected as a high‐depth/high‐quality germline control. The Exome Aggregation Consortium (ExAC) release 0.3 was used to filter out known polymorphic variants at a population frequency of <0.001 [30]. Variant calls generated for *BRAF* V600E, *NRAS* codon 61 and codon 12/13 as part of routine clinical work‐up were highly concordant with our mutation calls at a ratio of 96.5, 97.4 and 100% respectively (supplementary material, Figure S1).

### Identification of driver genes and genetic interactions

Non‐synonymous SNV and indels were analysed using dNdScv version 0.0.0.9 [31] to identify primary melanoma driver genes [false discovery rate (FDR)‐adjusted *P* value below 0.05]. The R package DISCOVER version 0.9.2 [32] was used to identify co‐occurring and mutually exclusive interactions. Pairwise tests were performed between all genes with an alteration frequency above 5% (*n* ≥ 26). Only known hotspot variants in *BRAF*, *NRAS* and *TERT* were considered. A 5% FDR threshold was applied, and gene pairs were excluded prior to analysis if co‐occurring mutations were found in ≥15% of tumours.

### RT‐PCR analysis of melanoma cell lines

To explore the expression of Transmembrane Phosphoinositide 3‐Phosphatase And Tensin Homolog 2 (*TPTE2*) in melanoma cell lines, we performed RT‐PCR (supplementary material, Figure S2). This analysis used the primers TPTE2_FWD: TGGTTTGTGCCCTCCTTATTGCC and TPTE2_REV: TCACATCATCATACAGAGGTGGACCG. QuantiTect RT Kit (Qiagen, Hilden, Germany) and AccuStart II PCR reagents (Quantabio, Beverly, MA, USA) were used with amplification involving 35 cycles (94 °C for 10 s, 62 °C for 15 s and 72 °C for 1 min).

### DNA somatic copy number alteration calling

Copy number calls were generated using ASCAT [33]. Only matched tumour‐normal pairs with mutation data were analysed. The mean tumour purity was 69%, and tumours estimated to be 100% pure were removed due to poor model fitting. A total of 401 tumour samples passed all filters. High‐level amplifications were classified as those with a total copy number ≥5 for diploid samples and ≥9 for tetraploid samples. Copy number events at the gene level were set using a strict filter: Only in cases where the whole gene was affected by the change was the event assigned to that gene.

### Gene dependency analysis

CRISPR genetic dependency data were downloaded from the Dependency Map (DepMap) portal [34] and reprocessed in‐house using CRISPRcleanR [35] and BAGELR to generate BAGEL lethality significance scores [35, 36]. These scores were binarized whereby positive BAGEL scores were assigned a score of 1 and negative BAGEL scores a score of 0. A Fisher's exact test was then used to test, on a gene‐by‐gene basis, the proportional frequency of lethality events among skin cancer cell lines compared to cell lines of all other tissue origins. Finally, the *P* values were adjusted for multiple testing using the FDR method. The analysis was identical to that described in Christodoulou *et al*. [37], except that we used data on all 29 melanoma cell lines released by the Broad Institute [34].

### Validation of interferon regulatory factor 4 dependency

Two cell lines defined as lethal by CRISPR screening (RVH421 and WM1799) and two non‐lethal (WM983B and HT144) melanoma cell lines were transfected with siRNAs. These siRNAs were ON‐TARGETplus SMARTpools (Dharmacon, Lafayette, CO, USA) designed against interferon regulatory factor 4 (*IRF4*), *ERH* (positive control/essential gene) or with a non‐targeting pool (negative control) according to the manufacturer's instructions. Cells were retransfected after 3 or 6 days and harvested for analysis after 10 days. Cells were stained with Annexin V‐PE (Biolegend, San Diego, CA, USA) and DAPI (Sigma‐Aldrich, St. Louis, MO, USA) and analysed by flow cytometry using a BD Fortessa II and FlowJo version 10 (Becton Dickinson, Franklin Lakes, NJ, USA). Successful gene knock‐down was confirmed by western blot analysis using an IRF4 antibody (Cell Signaling Technology, Danvers, MA, USA). GAPDH (Cell Signaling Technology, clone 14C10) or vinculin (Sigma‐Aldrich, clone V284) were used as loading controls. Cells were also analysed for c‐MYC protein expression using antibody clone Y69 (Abcam, Cambridge, UK).

## Results

### Somatic nucleotide landscape of primary melanoma

**Figure 1:**
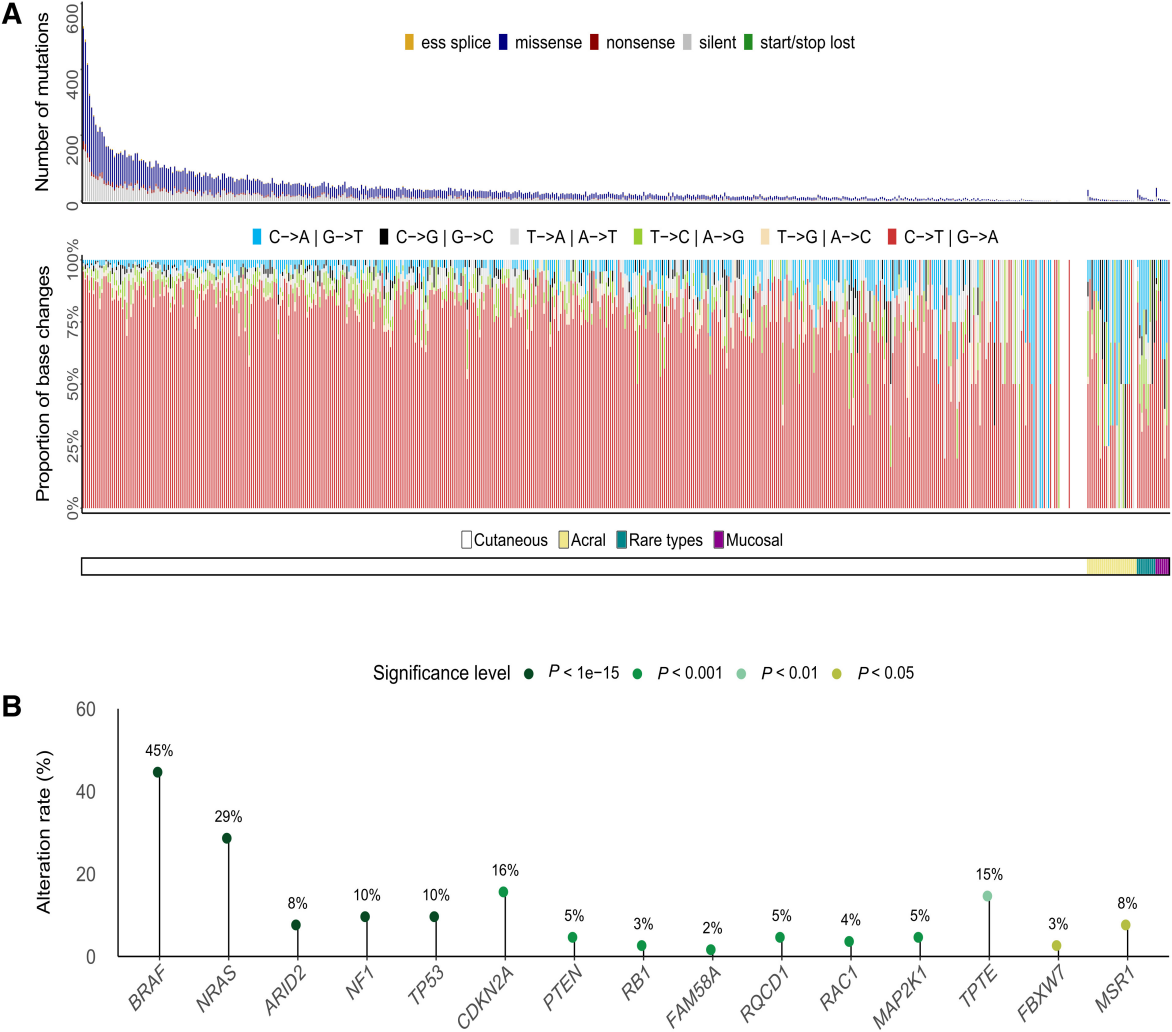
Summary of coding single nucleotide variants (SNVs) and driver genes in human primary melanoma (*n* = 524). (A) Overview of coding SNVs in primary melanoma. Top panel shows the number of exonic mutations in the capture regions and the distribution of variant consequences. Bottom panel shows the proportion of each base‐change type across the sample collection. (B) Primary melanoma driver genes identified using the algorithm DNdScv [31] and their respective alteration rate in the tumour collection.

We designed capture probes to sequence established cancer genes and genes implicated in melanoma development, as well as promoter regions. As part of this experiment, we also captured genomic regions to study copy number changes, genes implicated in the immune regulation of cancer and a collection of melanoma predisposition genes (supplementary material, Table S2). Our sample collection included 480 non‐acral cutaneous melanomas from sun‐exposed sites; 468 were sampled from the primary lesion, and 12 were sampled as nodal metastases where the primary lesion was unknown. A further seven mucosal, 24 acral and 13 cases of melanoma from rare sites, which were mainly tumours associated with the ano‐uro‐genital tract, were sequenced as a comparator (supplementary material, Table S1). All cases were derived from the LMC [21]. We sequenced both tumour and germline/blood DNA, and after duplicate removal, 76.3 and 89.6% of the targeted exome were covered at >30× respectively. For 465 cases we were able to generate paired tumour/germline sequence data. The 59 tumours without matched germline data were analysed as described in Materials and methods. After somatic variant calling, analysis revealed an average nonsynonymous mutation load of five mutations per megabase (MB) (SNVs and indels; range 0 to 72 mutations/MB) (see Materials and methods and supplementary material, Table [Link], [Link]). A high proportion of C > T base changes were found in almost all samples, the exception being acral and mucosal cases, as expected (Figure 1A) [16]. As reported previously, there was a statistically significant difference in the mutational load between cutaneous and other melanoma types (Kruskal–Wallis test; *P* < 0.001).

### Analysis of primary melanoma driver genes

Fifteen positively selected driver genes (FDR‐adjusted *P* value <0.05) were identified using dNdSCv (Figure 1B) [31], including the well‐established drivers *BRAF*, *NRAS*, *TP53* and *CDKN2A*. We also identified *FAM58A*, *RQCD1* and *MSR1*, which have recently been proposed as drivers [38, 39, 40] but were identified here *a priori* due to our large sample size and statistical power. Of note, our analysis also identified *TPTE* (Transmembrane Phosphatase With Tensin Homology), which has not been described as being associated with melanoma development previously but was identified in a pan‐cancer analysis of TCGA data [41] and further described as a driver using a machine‐learning approach of variants in genes from non‐unique regions of the genome [42]. *TPTE* is poorly expressed in normal tissue, with expression mainly confined to the testis; however, expression in melanomas and other cancers has been reported [43, 44, 45, 46]. Notably, multiple copies of *TPTE* pseudogenes appear to be localized to other chromosomes, including 13 and 22 [47], a feature known to potentially confound somatic variant detection, although *TPTE* genes were reported to be highly mutated in a recent pan‐cancer analysis that used long read technology for mutation confirmation [42]. The TPTE protein contains a PTEN C2 domain, and the mutations of *PTEN* and *TPTE/TPTE2* appear to be mutually exclusive (supplementary material, Figure S2). Intriguingly, despite their poor expression at the mRNA level, TPTE2 protein has been detected at the protein level in melanoma cell lines by western blotting, and *TPTE* and *TPTE2* have been scored as essential in melanoma cell lines by CRISPR screening [48], suggesting that they have a functional role in melanoma. Further, RT‐PCR revealed that *TPTE2* is expressed in two‐thirds of the melanoma cell lines tested (supplementary material, Figure S2).

### Coding and promoter hotspot mutations

**Figure 2:**
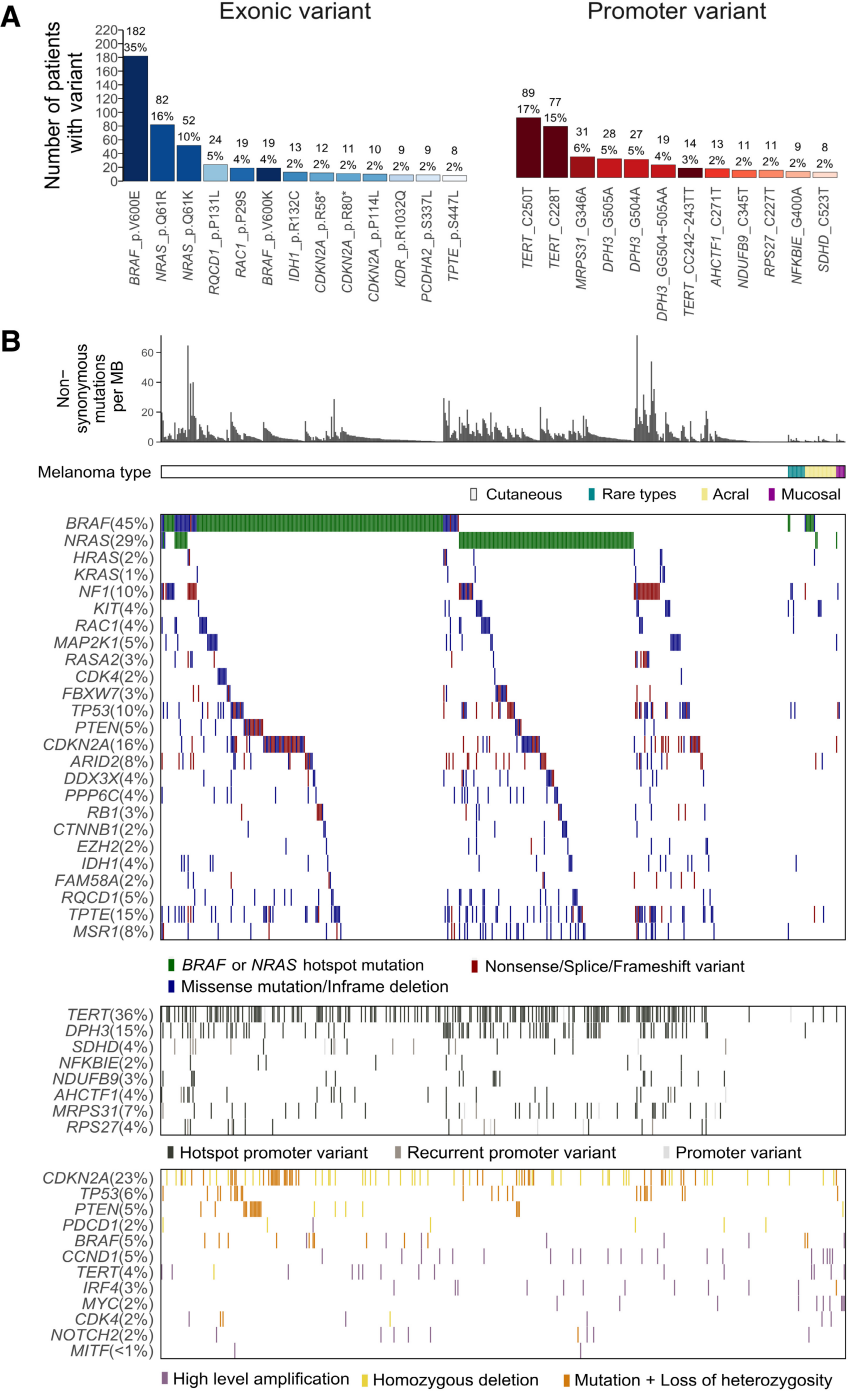
Mutational landscape of human primary melanoma (*n* = 524). (A) The most frequently mutated single nucleotide positions (exonic and promoter region). (B) Overview of the genetic landscape showing mutation load, non‐synonymous mutations in candidate driver genes, promoter mutations and copy number alterations. For SNVs, where there were multiple mutations in a gene in a case, the most pathogenic was plotted.

The most frequently mutated positions in our cohort of 524 primary melanomas included nucleotides in well‐known driver genes such as *BRAF*, *NRAS* and *TERT* (Figure 2A), and a similar frequency of *RQCD1* p.P131L mutations has been described in another primary melanoma cohort [39]. Hotspot mutations in *RQCD1*, *RAC1* and *IDH1* were primarily in non‐acral cutaneous melanomas. Similarly, the genes *PTEN*, *RAC1*, *RB1*, *DDX3X* and *PPP6C* were not mutated in acral or mucosal cases (Figure 2B), although fewer of these cases were sequenced as part of our cohort.

Notably, mucosal melanomas completely lacked *BRAF* hotspot mutations, and interestingly, no *TP53* mutations were found in acral melanomas. As previously described, hotspot mutations in *BRAF* and *NRAS* were mutually exclusive, with *BRAF* V600E mutations more frequently found in tumours arising on the trunk compared to the head (logistic regression, *P* value = 0.001, OR = 3.2) and in younger patients (logistic regression, *P* value = 0.003, OR = 0.98). Although tumours with *BRAF* V600K mutations showed a slightly higher mutational load compared to *BRAF* V600E tumours (logistic regression, *P* value = 0.0007, OR = 1.02), when compared to the *BRAF* wild‐type tumours, *BRAF* V600K tumours did not have significantly more somatic mutations (logistic regression, *P* value = 0.93, OR = 1.00) (supplementary material, Figure S3A).

We next explored non‐coding mutations where we noted a recurrent hotspot mutation in the promoter of the AT‐hook containing transcription factor 1 (*AHCTF1*) (GRCh37 chr1: 247095271) affecting 2% of melanomas (Figure 2A). This mutation may alter a highly conserved GABPA transcription factor binding site and is located in the UV‐damage‐associated sequence CTTCCG [49, 50], suggesting that it represents a position vulnerable to UV mutation. In the same way, recurrently mutated sites in the promoters of *DPH3*, *SDHD*, *NFKBIE*, *NDUFB9* and *MRPS31* (supplementary material, Figure S3B) were identified. These sites were previously reported as mutated in melanoma and carry the CTTCCG motif, which has been described as a binding site for ETS transcription factors [49]. Using data from the 507 immunotherapy‐naïve patients we were unable to find a significant association between mutational load and patient outcome (supplementary material, Figure S3C), a result in keeping with a recent discussion on the complexities of using tumour mutation burden as an immunotherapy response biomarker [51].

As discussed in what follows, we also successfully analysed the genome of 401 cases for copy number alterations, revealing that all cases with *TERT* promoter hotspot mutations (*n* = 180) were mutually exclusive from high‐level amplifications of *TERT* (*n* = 15), except for a single high‐mutation‐load tumour (supplementary material, Figure S3D).

### Landscape of genetic interactions in primary melanoma

**Figure 3:**
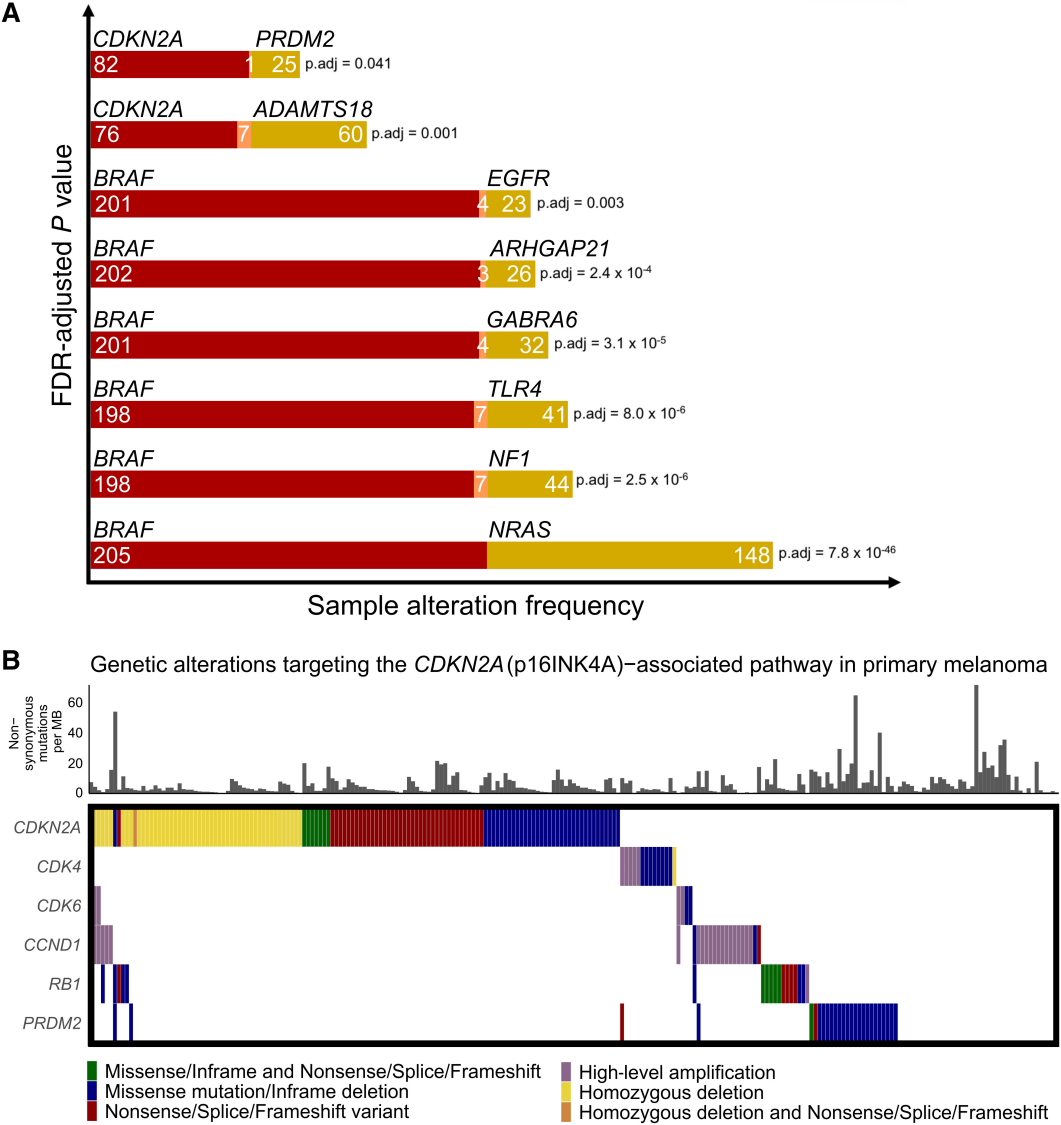
Mutually exclusive genetic interactions in human primary melanoma. (A) Gene pairs displaying a mutually exclusive alteration pattern. Samples with mutations in one gene in the pair are shown in red, the other gene in yellow, and samples with mutations in both genes are shown in orange. *P* values were calculated using the DISCOVER algorithm [32]. (B) Genetic alterations targeting important components of the *CDKN2A* (p16INK4A)‐associated regulatory pathway, including the newly discovered *CDKN2A*‐mutually exclusive gene *PRDM2*.

Since multiple genetic events are thought necessary to drive tumorigenesis, we used DISCOVER [32] to compute the presence of co‐occurring and mutually exclusive genetic interactions. This analysis revealed eight mutually exclusive gene pairs (FDR‐adjusted *P* value <0.05) (Figure 3A) but no co‐operating (synergistic) pairs of genes. Six genes had mutual exclusivity with *BRAF*, including the key driver genes *NRAS* and *NF1*, a pattern reflective of their complementary roles in activating the MAPK pathway. In the same way, *TLR4* and *EGFR* are both thought able to regulate the MAPK pathway [52, 53] and were also mutually exclusive with the mutation of *BRAF*. Notably, in colorectal cancer, BRAF inhibitors show limited efficacy, potentially due to the feedback activation of EGFR [54], whereas ectopic expression of EGFR in melanoma cells has been shown to elicit resistance to BRAF inhibitors [55] (supplementary material, Figure S4A).

Another mutually exclusive gene pair in our collection was *CDKN2A* and *PRDM2*. Although a similar trend was observed in samples from the TCGA SKCM cohort (ICGC release 28; 468 cases), this gene pair was not statistically significantly mutually exclusive because three samples in this collection had co‐occurring mutations. Since the TCGA SKCM dataset was smaller than our study, the DISCOVER analysis of the TCGA cohort had less power. *PRDM2* is a histone/protein methyltransferase that is capable of binding to the retinoblastoma protein [56], a binding partner that it shares with *CDKN2A*. Notably, genetic alterations of *PRDM2* were largely mutually exclusive from alterations of downstream components of the *CDKN2A* pathway (Figure 3B). Collectively, these findings suggest a possible role for *PRDM2* in processes related to *CDKN2A*, an observation that should be explored/validated functionally.

#### Somatic copy number alterations in primary melanoma

**Figure 4:**
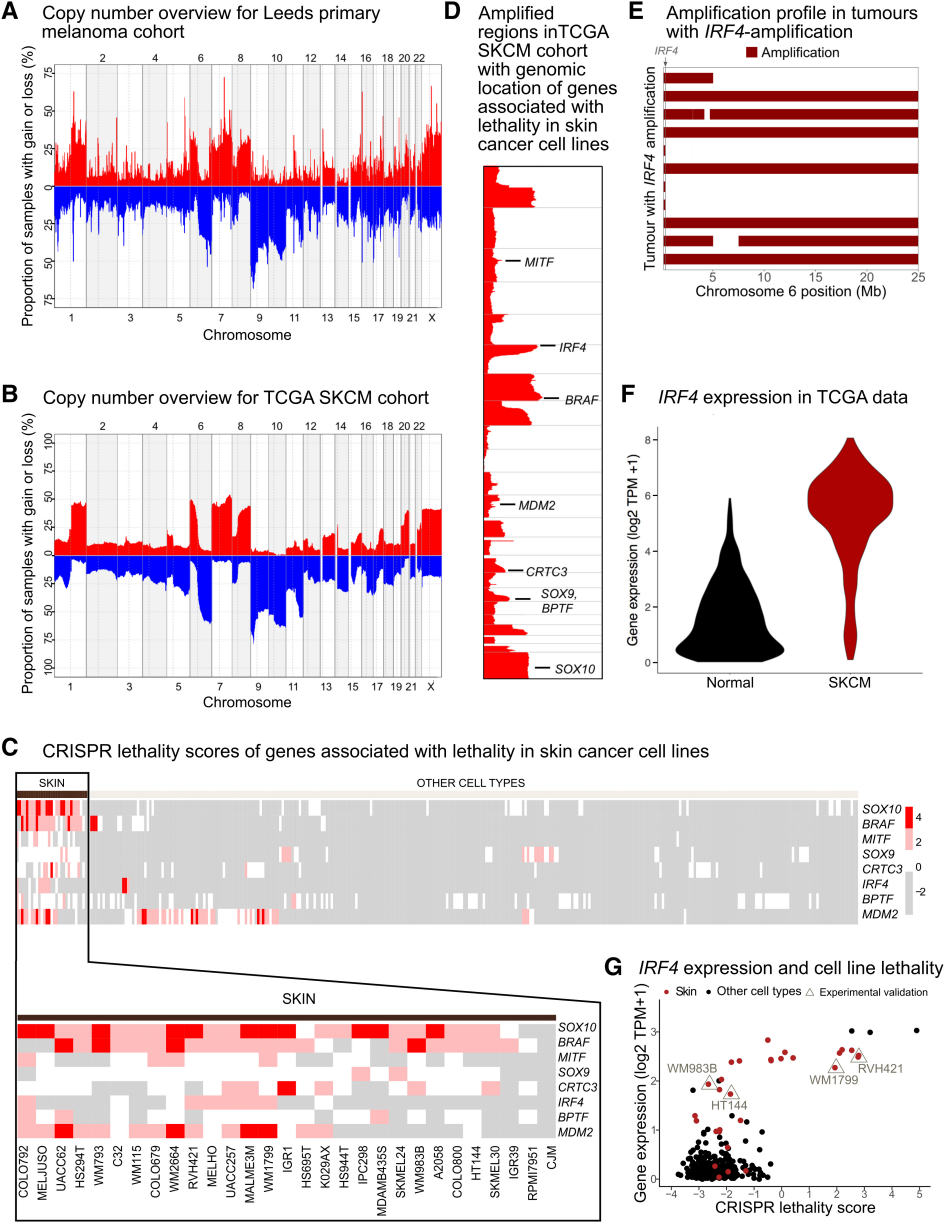
Whole genome copy number landscape and gene essentiality analysis. An overview of copy number alterations for (A) the human primary melanomas (Leeds melanoma cohort) [21] and (B) the TCGA SKCM dataset [17]. All segments with a copy number differing more than 0.6 from the sample average were used to generate the figures. Red illustrates gains and blue losses. (C) CRISPR lethality scores (higher scores correspond to a larger reduction in cell viability when the specific gene is silenced) of eight genes associated with lethality/reduced cellular fitness in skin cancer cell lines (Fisher's exact test, p‐adj < 0.01) versus all other cell lines in the Broad DepMap collection [34]. These data and the analysis approach used were described in detail in Christodoulou *et al*. [37]. Red indicates a negative effect of CRISPR on cancer cell line fitness. Note that grey does not indicate an effect on cell fitness because the Bayes algorithm used is not configured to identify effects that enhance cell growth (see Materials and methods). (D) Amplified regions in the TCGA SKCM cohort overlaid with the genomic location of the eight genes associated with lethality in skin cancer cell lines. (E) 25‐Mb regions of high‐level amplifications of chromosome 6, shown for all *IRF4*‐amplified samples. The location of *IRF4* is shown in grey. (F) Expression of *IRF4* in the Rahman *et al*. [57], reprocessed TCGA expression dataset. (G) Correlation between *IRF4* expression and cell line CRISPR lethality scores.

Copy number data were generated using ASCAT [33] for 401 samples, where sequence coverage and data quality were sufficient (Figure 2B, see Materials and methods). Frequently amplified regions included chromosomes 1q, 6p, 7 and 8q, whereas chromosomes 6q, 9 and 10 were more commonly deleted (Figure 4A and supplementary material, Table S6). We compared the copy number profiles of our primary melanomas to the TCGA melanoma cohort (TCGA SKCM) [17] and found remarkably similar profiles (Figure 4B), suggesting these genetic events manifest at an early stage of tumour development. Amplification of chromosomes 1q, 6p and 8q was particularly pronounced in mucosal and, to some extent, acral melanoma samples (supplementary material, Figure S4B). We observed that some driver genes were more often targeted by mutations in non‐acral cutaneous melanomas, whereas the same genes in acral and mucosal melanoma were more often altered by copy number events (supplementary material, Figure S4C).

At the gene level, *CCND1* was the most common high‐level amplification, found in 20 samples (5% of all melanomas) (Figure 2B). Amplifications targeting *MYC* on chromosome 8q were found in 10 samples (2%), being more frequent in acral (three samples, 15%), mucosal (three samples, 50%) and rare (one sample, 11%) subtypes compared to non‐acral cutaneous tumours (three samples, <1%). *TERT* was found amplified in 15 samples (4%), and *IRF4*, located on chromosome 6p, was amplified in 11 (3%) samples. Deletion of *CDKN2A* was the most common copy number event in primary melanoma: 63% of samples showed copy number losses affecting *CDKN2A*, with 13% showing homozygous deletions across the entire gene.

### Essentiality of primary melanoma drivers

**Figure 5:**
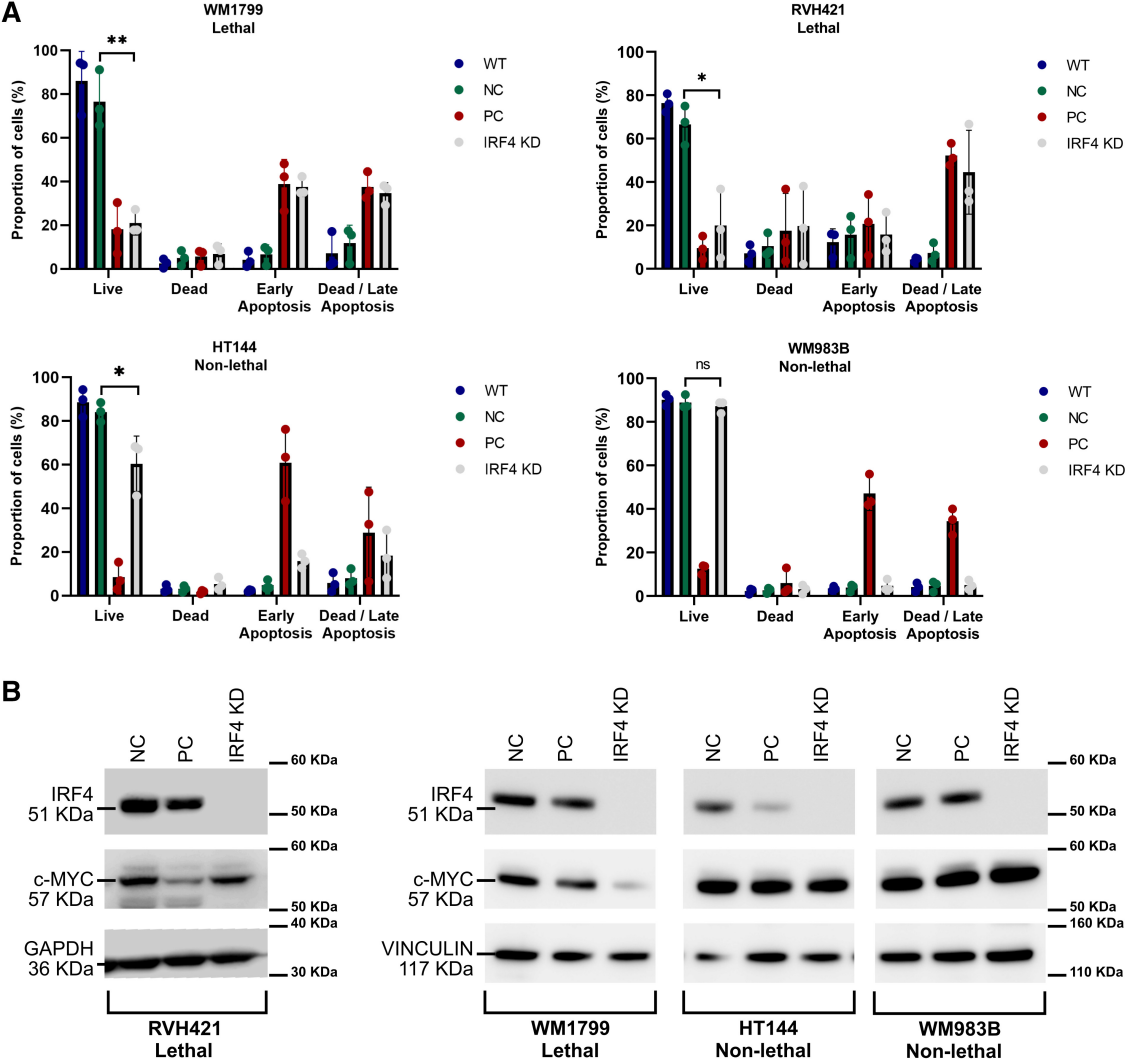
Validation of the essentiality of *IRF4* in melanoma cell lines. (A) siRNA‐mediated knockdown of *IRF4*. WM1799 and RVH421 are cell lines identified by CRISPR screening as being sensitive to *IRF4* loss, whereas HT144 and WM983B were not scored in this way. WT, NC, PC and IRF KD refer to siRNA treatments and correspond to untransfected, negative control, positive control and IRF4 siRNAs, respectively. More details are provided in Materials and methods. These data were collected from three independent experiments. Analysis was performed using a Student's two‐tailed *t*‐test comparing the number of viable/live cells between the *IRF4* siRNA transfected cells versus cells transfected with the negative control siRNA. *P < 0.05, **P < 001. (B) Analysis of protein lysates for IRF4 and c‐MYC and for expression of the loading controls GAPDH/VINCULIN. These experiments are representative of three independent experiments.

We next used Broad DepMap data where CRISPR‐Cas9 screening had been used to identify essential genes in 342 cancer cell lines, 29 of which were melanoma lines. Following correction for copy number–associated artefacts using CRISPRCleanR [35], analysis of these data revealed 35 genes as selectively more likely to be essential in melanoma cell lines versus the other 313 tumour cell lines derived from 22 other tissue types. By intersecting these 35 genes with those genes in amplified regions of the melanoma genome, reasoning that these regions may contain oncogenes selected for during tumour evolution, we identified eight genes whose amplification appeared to create a melanoma‐specific vulnerability (Fisher's exact test, FDR‐adjusted *P* value <0.01). Among these eight genes were important melanoma genes such as *BRAF*, *MITF*, *MDM2* and *SOX10* (Figure 4C,D). Notably, we also found *IRF4*, located in a highly amplified region of chromosome 6p, to be essential in cell lines that expressed high levels of the gene (Figure 4A–G). These tumours were targeted by a mixture of broad and focal amplifications (Figure 4E). Additionally, we found increased expression of *IRF4* in melanoma tumours compared to non‐cancerous tissues from a range of tissue sites (Figure 4F), suggesting *IRF4* amplification could play a role in melanoma development. Other cancers, such as multiple myeloma, have been shown to be dependent on IRF4, and in this context depletion of *IRF4* or treatment with an IRF4 inhibitor has been shown to induce lethality [58]. We therefore hypothesize that some melanoma cells may upregulate *IRF4* and develop a dependency on this transcription factor such that it may represent a vulnerability that could be exploited therapeutically. We next validated the requirement of melanoma cells for *IRF4* using the orthogonal technology of siRNA knockdown and flow cytometry to assess viability. Analysis in this way revealed, as observed with CRISPR, that depletion of *IRF4* results in reduced cancer cell fitness associated with cell death and apoptosis (Figure 5A and supplementary material Figure S5). Notably, these phenotypes appeared independent of changes in MYC protein expression (Figure 5B), an established downstream target of IRF4, suggesting MYC‐independent or a pleiotropic role for IRF4 in melanoma cell survival.

## Discussion

In this study, we profiled the genomic landscape of primary melanomas as a complement to previous large‐scale efforts that primarily focused on metastatic disease. Understanding primary melanoma is critically important for clinical practice because the vast majority of patients present at this stage and understanding the aetiology of these cancers may help identify those patients who would benefit most from adjuvant therapies. Our analysis revealed 15 driver genes that were significant following dNdScv analysis. Our large study was able to confirm *FAM58A*, *RQCD1* and *MSR1* as statistically significant driver genes (Figure 1). *FAM58A* is a cyclin family member, implicated in the regulation of the actin network and ciliogenesis [59], whereas both *RQCD1* and *MSR1* are poorly studied genes implicated in the CCR‐NOT and PI3K/AKT pathways, respectively [60, 61]. Our analysis also revealed *TPTE*, a gene with multiple related pseudogenes, as another candidate, although further studies will be required to validate this observation. Using DISCOVER analysis [32] we identified eight statistically significant mutually exclusive genetic interactions, six with *BRAF* and two with *CDKN2A* (Figure 2, 3), each representing important insights into the pathways through which melanoma develops. With this method, which accounts for individual tumour mutation rates, it was notable that we failed to identify any genes that were statistically significantly co‐mutated. This might suggest that there are multiple cooperative interactions consisting of lots of gene pairs, and despite analysing 524 tumours, we still lacked the power to detect these. It is also possible that non‐genetic mechanisms or genes not studied here cooperate with driver events, with these interactions yet to be discovered. Similarly, as suggested previously [32], an alternative hypothesis could be that the biology of melanoma is similar to that of other cancers where biology drives mutual exclusivity, for example activation of the MAPK pathway via either *BRAF* or *NRAS* mutation, but chance explains most co‐occurrences. The gene *PRDM2* showed a mutation pattern of mutual exclusivity with *CDKN2A* and is of particular interest since PRDM2 is a binding partner of RB1, a well‐established melanoma driver [62, 63]. Finally, by intersecting copy number analysis of our tumour collection with genome‐wide CRISPR dependency data, we revealed a potential vulnerability driven by amplification of the transcription factor *IRF4*, a result with implications for our understanding of melanoma biology.

## Author contributions statement

The majority of the computational analysis was performed by SB with assistance from MR, JPL, IM, KH, VI, UM, CDRE, PVL, MLK and DTB. DTB and J N‐B led the Leeds Melanoma Cohort Study with assistance from MH and TM. Experiments with cell lines were performed by SB, LSASM, PAP and SSME. The experiments were designed by SB and DJA who wrote the paper, which was approved by all authors.

## Supporting information


**Figure S1.** Analysis of known gene mutations
**Figure S2.** Analysis of *TPTE* gene family
**Figure S3.** Analysis of genetic landscape of melanoma
**Figure S4.** MAPK pathway mutual exclusivity and analysis of copy number landscape in this cohort
**Figure S5.** Functional analysis of *IRF4* loss in melanoma cell lines

**Table S1.** Clinical characteristics of patients in Leeds melanoma cohort, divided into respective subtypes

**Table S2.** Targeted capture bait design regions

**Table S3.** Summary of all mutation calls made in this study including those in non‐coding regions

**Table S4.** Summary of all mutation calls made in this study that were protein changing and in melanoma genes

**Table S5.** Summary of all indel calls made in this study

**Table S6.** Summary of copy number events that alter whole cancer/melanoma genes

## Data Availability

The raw sequencing data are available for download from the European Genome‐phenome Archive (dataset ID EGAD00001008360).
