# Supplementary material for: Mutually exclusive genetic interactions and gene essentiality shape the genomic landscape of primary melanoma
Source: J Pathol. 2022 Nov 9;259(1):56–68. doi: 10.1002/path.6019 (PMC10098817; doi:10.1002/path.6019)
Supplement: Supplementary file 1 — Figure S1. Analysis of known gene mutations Figure S2. Analysis of TPTE gene family Figure S3. Analysis of genetic landscape of melanoma Figure S4. MAPK pathway mutual exclusivity and analysis of copy number landscape in this cohort Figure S5. Functional analysis of IRF4 loss in melanoma cell lines [file PATH-259-56-s007.docx]

**Mutually exclusive genetic interactions and gene essentiality shape the genomic landscape of primary melanoma**

S Birkeälv *et al. J Pathol* DOI: <https://doi.org/10.1002/path.6019>

**Supplementary Figures S1–S5**

Reference numbers refer to the main text list


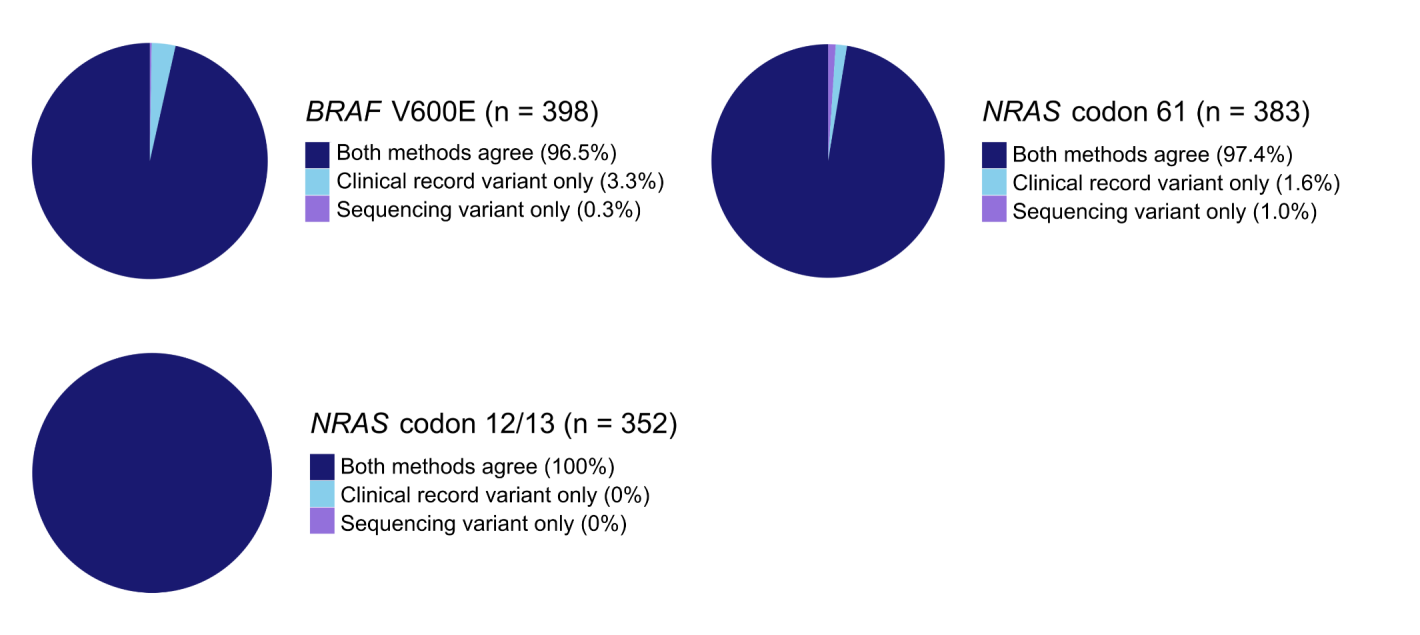


**Figure S1. Analysis of known gene mutations.** Variant calling concordance with participant clinical records for *BRAF* and *NRAS* mutation status. More details can be found in Materials and methods.


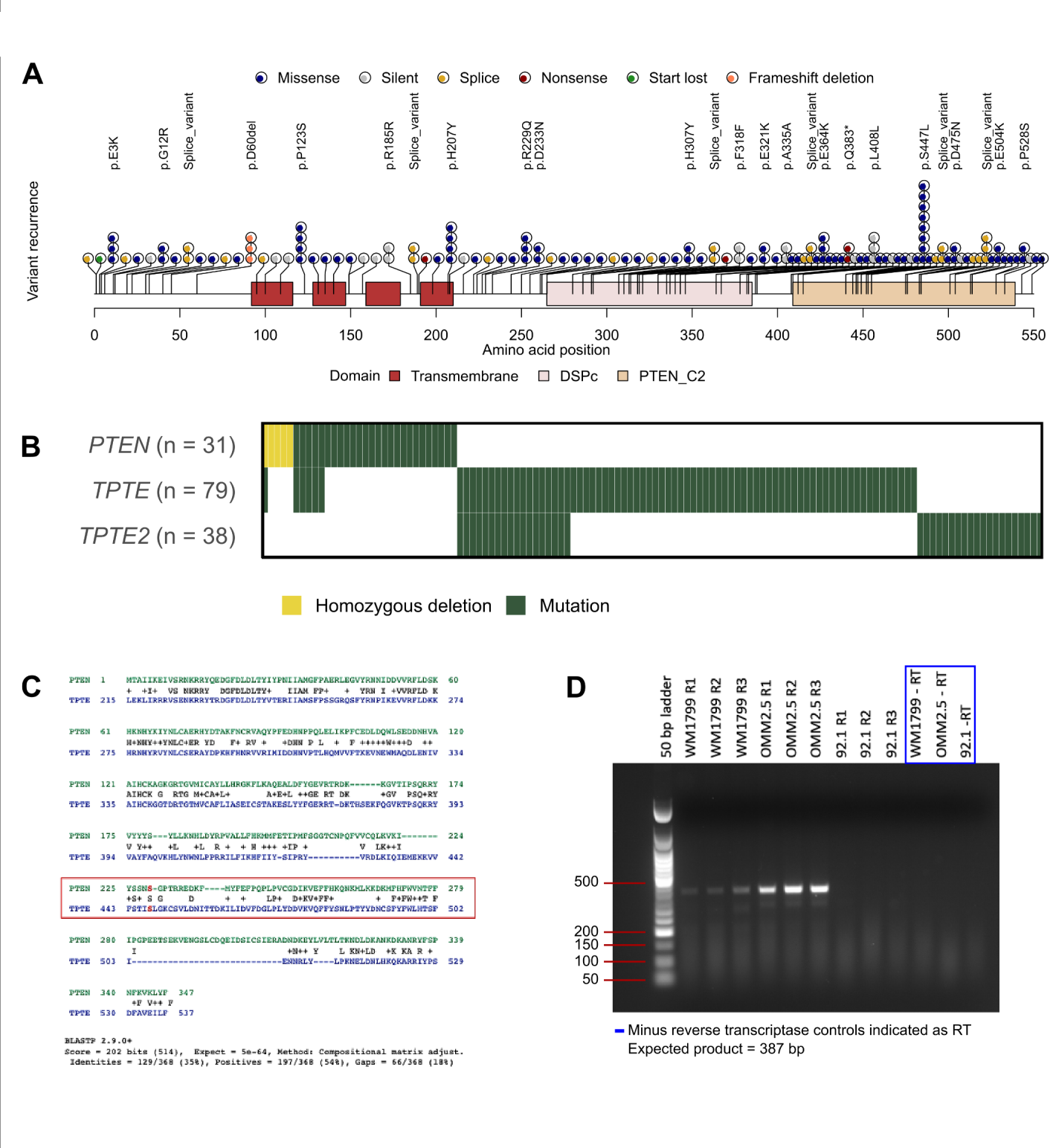


**Figure S2. Analysis of the *TPTE* gene family.** (A) Distribution of mutations in the TPTE protein in primary melanomas (n = 524) (B) Pattern of mutations and homozygous deletions between the paralogues *PTEN*, *TPTE* and *TPTE2*. (C) Pair-wise protein BLAST analysis comparing TPTE to PTEN suggests the position of the novel TPTE hotspot S447L variant corresponds to position S229 in PTEN (Red residue). (D) RT-PCR showing expression of *TPTE2* in human melanoma cell lines. For experimental details see Materials and methods.


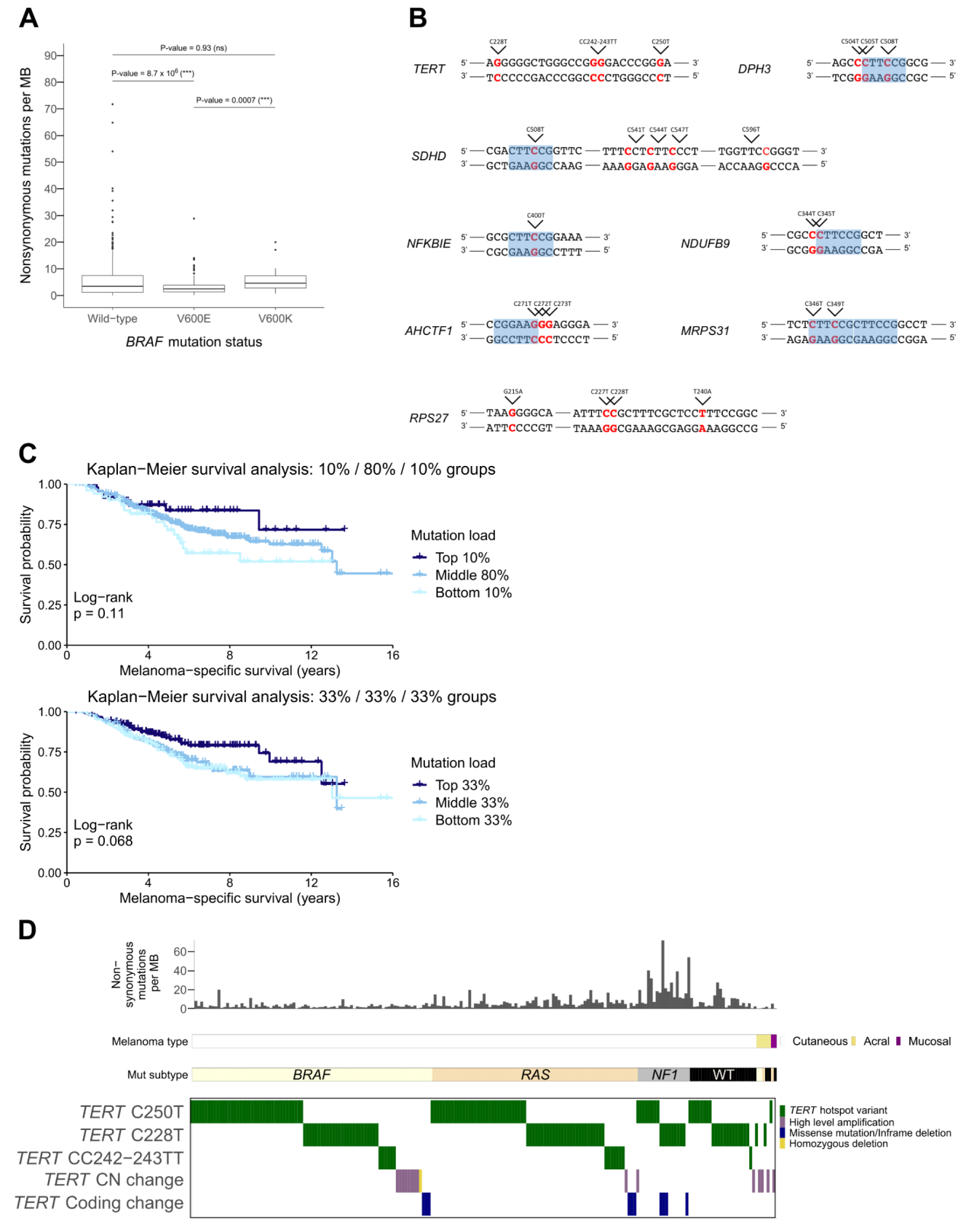


**Figure S3. Analysis of the genetic landscape of melanoma.** (A) Mutation load of tumours with *BRAF* V600E or *BRAF* V600K mutations and those that are wildtype for mutations in the V600 codon. Statistical significance was calculated using linear regression (univariate). (B) ETS transcription factor consensus motif pattern among recurrent promoter mutations in melanoma. Recurrent variant positions are marked red, the CTTCCG UV damage-associated pattern is highlighted in blue [49]. (C) The effect of mutational load on patient outcome. These analyses were performed using the 507 immune therapy naïve patients. (D) Genetic alterations targeting the *TERT* gene, and the corresponding mutational load and tumour subtypes.


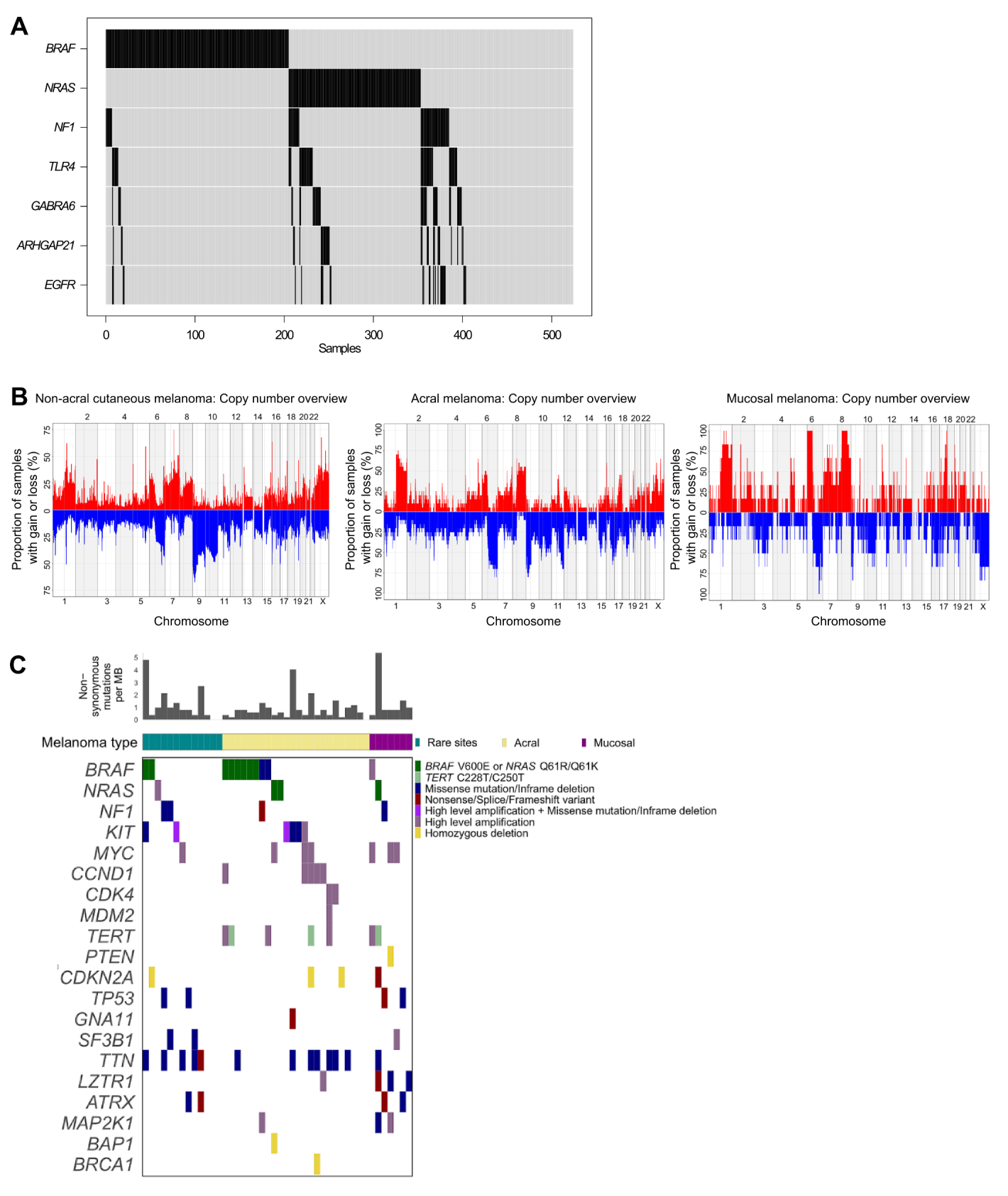


**Figure S4. MAPK pathway mutual exclusivity and analysis of the copy number landscape in this cohort.** (A) Mutual exclusivity of the MAPK pathway. (B) Genome-wide overview of copy number alterations across different melanoma subtypes. (C) Genetic alterations in rarer subtypes of melanoma.


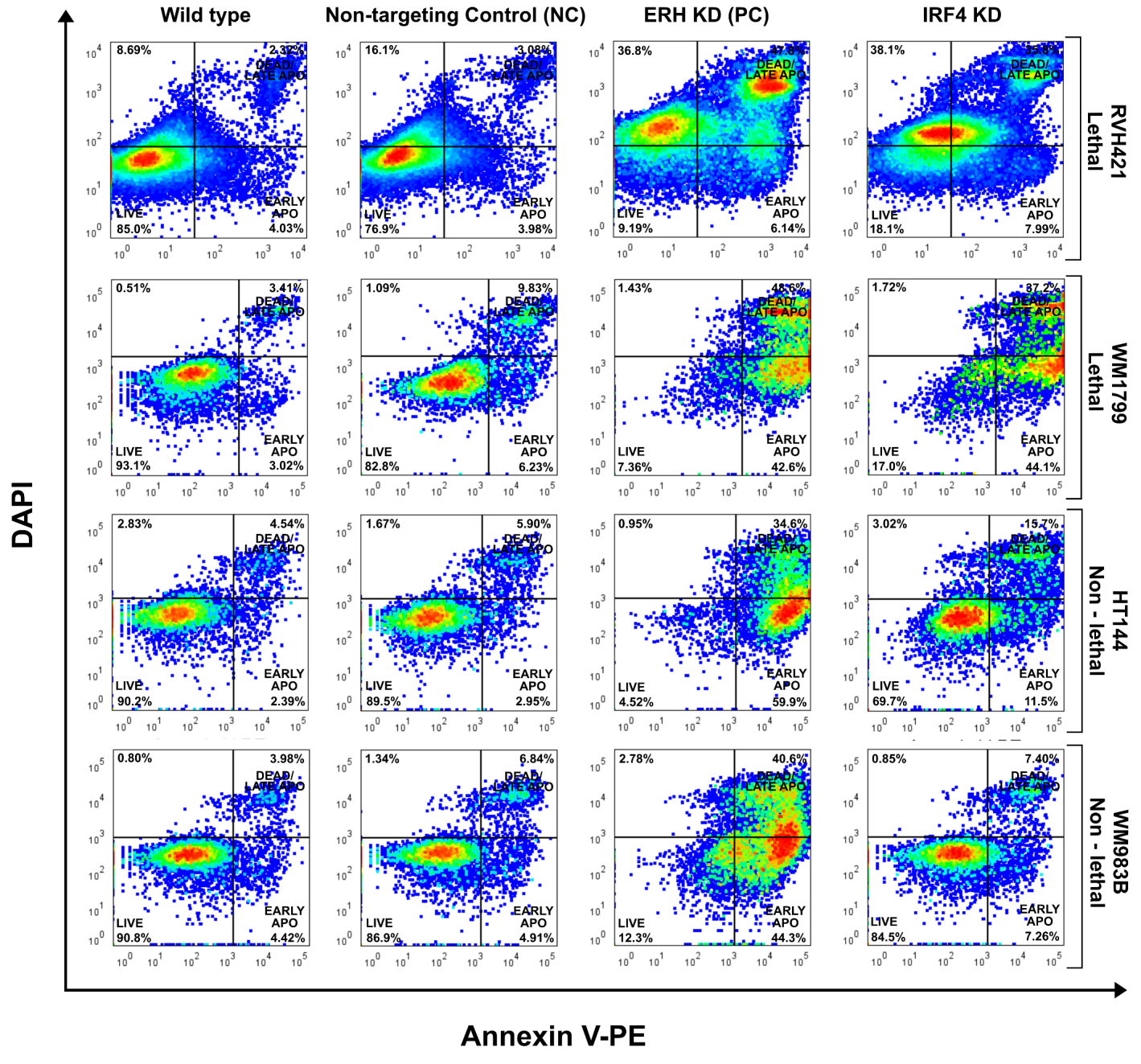


**Figure S5. Functional analysis of *IRF4* loss in melanoma cell lines.** Analysis of cell viability by use of DAPI and Annexin staining. This experiment involved four cell lines. RVH421 and WM1799 were defined as requiring *IRF4* by CRISPR screening, while HT144 and WM983B were not (see materials and methods). This result was replicated on three separate occasions (See Figure 5). Wild type represents untransfected cells, NC and PC are non-targeting and positive controls, respectively. IRF4 knockdown (KD) was achieved using a pool of siRNAs (see materials and methods). The top right quadrant represents dead cells or late apoptosis, the bottom right early apoptosis, while the bottom left quadrant are live cells.
