# Supplementary material for: Mutually exclusive genetic interactions and gene essentiality shape the genomic landscape of primary melanoma
Source: J Pathol. 2022 Nov 9;259(1):56–68. doi: 10.1002/path.6019 (PMC10098817; doi:10.1002/path.6019)
Supplement: Supplementary file 2 — Table S1. Clinical characteristics of patients in Leeds melanoma cohort, divided into respective subtypes [file PATH-259-56-s005.docx]

**Mutually exclusive genetic interactions and gene essentiality shape the genomic landscape of primary melanoma**

S Birkeälv *et al. J Pathol* DOI: <https://doi.org/10.1002/path.6019>

**Table S1.** Clinical characteristics of patients in the Leeds melanoma cohort, divided into respective subtype.

|  | **Overall (n=524)** | **Cutaneous (n=480)** | **Acral (n=24)** | **Mucosal (n=7)** | **Ano-uro-genital (n=13)** |
| --- | --- | --- | --- | --- | --- |
| **Sex**  - Female | 263 (50%) | 236 (49%) | 11 (46%) | 7 (100%) | 9 (69%) |
| - Male | 261 (50%) | 244 (51%) | 13 (54%) | 0 (0%) | 4 (31%) |
| **Age (years)** |  |  |  |  |  |
| - Mean (SD) | 57 (±12) | 57 (±12) | 56 (±12) | 63 (±8.7) | 60 (±13) |
| **Stage^a^** |  |  |  |  |  |
| - I | 167 (32%) | 162 (34%) | 4 (17%) | 0 (0%) | 1 (8%) |
| - II | 253 (48%) | 226 (47%) | 18 (75%) | 4 (57%) | 5 (38%) |
| - III | 97 (19%) | 88 (18%) | 2 (8%) | 1 (14%) | 6 (46%) |
| **Breslow thickness^a^ (mm)** |  |  |  |  |  |
| - Mean (SD) | 3.0 (±2.4) | 2.9 (±2.2) | 3.6 (±2.2) | 3.5 (±0.87) | 7.1 (±5.4) |
| **Ulceration^a^** |  |  |  |  |  |
| - No | 289 (55%) | 281 (59%) | 7 (29%) | 0 (0%) | 1 (8%) |
| - Yes | 169 (32%) | 138 (29%) | 17 (71%) | 4 (57%) | 10 (77%) |
| **Mitotic rate^a^ (mitoses/ per mm^2^)** |  |  |  |  |  |
| - <1 | 66 (13%) | 61 (13%) | 3 (12%) | 1 (14%) | 1 (8%) |
| - >=1 | 402 (77%) | 365 (76%) | 20 (83%) | 6 (86%) | 11 (85%) |
| **Tumour-infiltrating lymphocytes^a^** |  |  |  |  |  |
| - Absent | 83 (16%) | 70 (15%) | 9 (38%) | 2 (29%) | 2 (15%) |
| - Yes (Unclassified) | 47 (9%) | 46 (10%) | 0 (0%) | 0 (0%) | 1 (8%) |
| - Non-brisk | 215 (41%) | 195 (41%) | 12 (50%) | 3 (43%) | 5 (38%) |
| - Brisk | 77 (15%) | 77 (16%) | 0 (0%) | 0 (0%) | 0 (0%) |
| **Mutational subtype** |  |  |  |  |  |
| - *BRAF* | 205 (39%) | 197 (41%) | 6 (25%) | 0 (0%) | 2 (15%) |
| - *NRAS* | 148 (28%) | 145 (30%) | 2 (8%) | 1 (14%) | 0 (0%) |
| - *NF1* | 32 (6%) | 28 (6%) | 1 (4%) | 1 (14%) | 2 (15%) |
| - WT | 139 (27%) | 110 (23%) | 15 (62%) | 5 (71%) | 9 (69%) |
| **Mutation load^b^** |  |  |  |  |  |
| - Mean (SD) | 5.1 (±7.2) | 5.5 (±7.5) | 0.83 (±0.83) | 1.5 (±1.7) | 1.2 (±1.3) |
| **Relapse** |  |  |  |  |  |
| - No | 333 (64%) | 313 (65%) | 14 (58%) | 2 (29%) | 4 (31%) |
| - Yes | 191 (36%) | 167 (35%) | 10 (42%) | 5 (71%) | 9 (69%) |
| **Immunotherapy (post biopsy)** |  |  |  |  |  |
| - No | 507 (97%) | 466 (97%) | 23 (96%) | 6 (86%) | 12 (92%) |
| - Yes | 17 (3%) | 14 (3%) | 1 (4%) | 1 (14%) | 1 (8%) |
|  |  |  |  |  |  |

^a^Missing values have been excluded from the table output.

^b^Mutation load is defined as the number of nonsynonymous mutations per megabase (MB) of genomic space sequenced.
